# Off-Axis Compressive Behaviour of Fibre Reinforced Thermoplastic Composites

**DOI:** 10.3390/ma15165547

**Published:** 2022-08-12

**Authors:** Yifan Ma, Yazhi Li, Lu Liu

**Affiliations:** 1 Department of Aeronautical Structural Engineering, School of Aeronautics, Northwestern Polytechnical University, Xi’an 710072, China; 2 Department of Aircraft Design, School of Aircraft, Xi’an Aeronautical Institute, Xi’an 710077, China

**Keywords:** thermoplastic composites, off-axis compression, fibre kinking, failure envelope, fracture angle, DIC technique

## Abstract

This paper presents an experimental investigation on the mechanisms of damage onset and evolution in unidirectional PEEK/AS4 carbon fibre reinforced thermoplastic (CFRTP) composites subjected to off-axis compressive loadings. A test fixture was designed to prevent buckling, splitting, and end collapsing of the specimens during the test. A series of compression tests were conducted with specimens of various off-axis angles. The displacement and strain fields of all specimens during the tests were evaluated by the digital image correlation (DIC) method. In combination with the DIC results, the influence of the off-axis angles on the failure mechanisms and nonlinear stress–strain responses were analysed. The corresponding failure mechanisms were examined by scanning electron microscopy (SEM). The fracture angles of the tested specimens were evaluated and analysed according to Puck’s theory. The off-axis compression failure envelope based on LaRC05 and Hashin criteria was presented and compared to the experimental results. It was shown that the LaRC05 criterion can provide accurate predictions when the off-axis angle is larger than 15°. The complex failure mechanisms were analysed to better understand the effect of ductility of the thermoplastic matrix to the composites. The series of tests provide an experimental failure envelope in combined stress states and can be used for the evaluation of failure theories and the criteria of thermoplastic composites.

## 1. Introduction

The increasing application of carbon fibre-reinforced thermoplastic composites (CFRTPs) in aerospace engineering has drawn significant attention to the compressive failure of unidirectional laminates, which is generally recognised as a combined result of various mechanisms. It is one of the most challenging tasks to comprehensively describe these behaviours. A considerable amount of research on off-axis compression tests has been conducted in the past decades. Failure modes such as fibre splitting, interface decohesion, fibre kinking, and matrix shear failure have been experimentally observed and further explained in several theoretical studies [1,2,3]. The fibre kinking is the compressive fracture mechanism of the fibre reinforced composites (FRP), which is caused by the micro-buckling of the fibres. This failure mode was reported by several researchers [4,5]. Budiansky and Fleck [6] took the strain hardening effect of the matrix into consideration when analysing the fibre kinking. Lee and Waas [7] studied the compressive strength and failure modes of a unidirectional glass fibre reinforced polymer (GFRP) and carbon fibre reinforced polymer (CFRP). A finite element (FE) model was presented to study the effect of initial misalignment angles of the fibres. Pimenta et al. [2] studied the initiation and propagation of kink-bands through compression tests of notched unidirectional CFRP plates. Ueda et al. [8] investigated the kink-band formation of CFRP using X-ray micro-CT. The failure model proposed by Pinho et al. [9] included the fibre kinking theory, which could accurately predict the compressive failure of the fibre reinforced unidirectional composite. The splitting was initially observed by Piggott and Harris [10] through a compression test of the unidirectional GFRP composites. Later, splitting was identified in the compressive failure mechanisms of the carbon fibre reinforced composites by Oguni and Ravichanran [11]. Lee and Waas [7] investigated the effect of different fibre volume fractions on the compressive failure mechanisms of unidirectional glass fibre and carbon fibre composites. A combined fibre kinking and splitting failure mode was reported at different fibre volume fractions. Prabhakar et al. [12] studied the relationship between fibre kinking and fibre splitting of unidirectional composites and concluded that the splitting mode dominated the compressive strength. Yerramalli et al. [13] investigated the fracture mechanics of glass fibre and carbon fibre composites under combined compressive and shear stresses, and suggested that both matrix splitting and fibre kinking contributed to the composite failure.

The compressive strength of the unidirectional composite is also affected by transverse shear behaviour [14]. The mingled shear stress during the compression of unidirectional CFRP composites was investigated by Vogler et al. [14]. Their experiments showed that the shear field introduces extra fibre rotation and bending. Tsai and Sun [15] conducted off-axis compressive tests of CFRP specimens under different strain-rates. In-plane shear stress was extracted from the compounded stress field of the off-axis compression. Their results showed that the in-plane shear strength plays a significant role in the specimen failure.

Several models [16,17] based on physical mechanisms were proposed to reflect the different failure modes and correlate well with the experimental phenomena. Zhou et al. [18] investigated the failure mechanism of GFRP composites under off-axis compressive loading conditions. Fibre kinking, fibre buckling, and matrix cracking were observed in the specimens with different off-axis angles. González and Llorca [19] investigated the transverse compression mechanisms of the unidirectional GFRP and CFRP composites. Micromechanical simulation indicated that the interface decohesion and shear failure were the two main failure modes. Matsuo and Kageyama [20] developed a modified kink band model that considered transverse tension and shear stresses within the kink band area. Thomson et al. [21] conducted 0°, 3°, 6°, 10°, and 15° off-axis compression experiments using unidirectional and cross-ply IM7/8852 specimens with different designs. The results suggest that the unidirectional dog-bone specimens will cause stress concentration at the changing area. Lee and Soutis [22] evaluated the compressive strength of unidirectional CFRP composites using compression fixtures that clamped both ends of the specimens. The results showed that the over clamping of specimens and stress concentration at the gripping area had a severe effect on the compression strength. Misaligned fibres and wavy plies also affected the compressive failure stress. However, the mechanical behaviour of unidirectional composites under compression, which is vital for understanding the fibre kinking and splitting mechanics, has not been investigated thoroughly due to the difficulty of conducting the experiment. Thus, to obtain reliable measurements of off-axis compression strength and investigate the failure mechanisms of unidirectional composites, carefully designed experiments need to be conducted.

The purpose of this paper is to (1) develop a suitable off-axis compression test fixture and specimen configuration; (2) study the compressive behaviour of PEEK/AS4 carbon fibre reinforced thermoplastic composite through off-axis compression test, which would produce a set of combined stress states with uniaxial load and lead to different failure modes; (3) investigate the influence of the fibre orientation angles on the mechanical behaviour of thermoplastic composites; (4) provide experimental evidence to develop the failure envelope of thermoplastic composites; and (5) analyse the failure modes of unidirectional thermoplastic composites under combined stress states to evaluate the mechanism-based failure criteria for thermoplastic composites.

## 2. Materials and Methods

The material used in this study was Cetex^®^ TC1200 PEEK/AS4 thermoplastic/carbon prepreg produced by Toray Advanced Compsites, Nottingham, UK. PEEK (polyetheretherketone) is a semi-crystalline, high-performance engineering thermoplastic. Thermoplastic materials have higher toughness and ductility compared to thermosets. PEEK has a good toughness even among thermoplastic polymers.

The nominal thickness of the Cetex^®^ TC1200 PEEK/AS4 prepreg was 0.14 mm. The nominal fibre volume fraction was 34%. The fibre diameter was 7 μm. The overall density of the prepreg was 1.46 g/cm^3^. The primary properties of the PEEK/AS4 prepreg is summarized in Table 1.

### 2.1. Fabrication Method

A 400 × 700 mm^2^ unidirectional laminate was manually stacked and fabricated using the RYJ-600Z1 hot press machine manufactured by the SHANGHAI XINNUO INSTRUMENT GROUP in Xi’an, China. The laminate was consolidated in the hot press compression chamber at 390 °C for 20 min and then cooled down at 4 °C/min until room temperature.

To study the effect of fibre orientation angles on the failure mechanisms, the fibre orientation angles with respect to the loading direction were selected as θ = 0°, 15°, 30°, 45°, 60°, 75°, and 90°.

**Figure 1:**
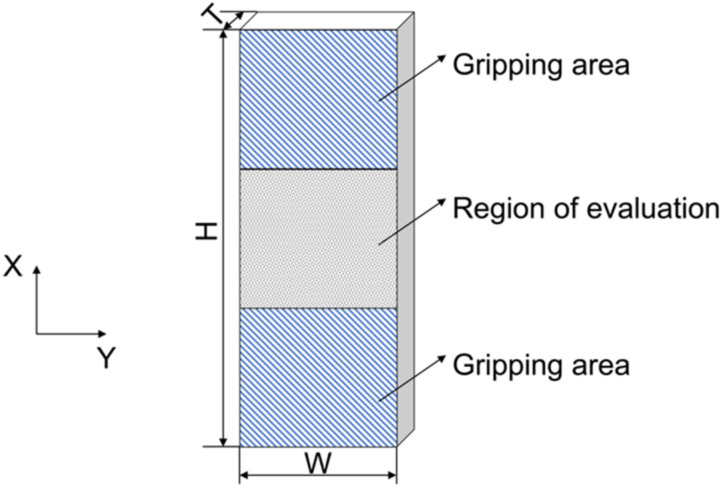
A schematic of the off-axis specimens.

The sketch of the off-axis compression test specimen is shown in Figure 1. Nominal dimensions of the specimens were 36 mm (H) × 12 mm (W) × 3 mm (T). The area of the evaluation section was 12 mm × 12 mm. The specimen was designed to be thick in relation to the specimen length to improve the stability. The off-axis specimens were cut from the laminated plate using a HEAD2015BA waterjet machine manufactured by HEAD in Shenyang, China.

To prevent slipping, the loading surfaces of every specimen were polished to ensure that they were flat, parallel to each other, and perpendicular to the loading direction. This process was completed on a CVMP-2 grinding and polishing machine manufactured by XIWAKA in Dongguan, China. The grinding of samples was performed with SiC powder. The powder sizes used were 220, 400, 600, 800, 1200, and 1500 per stage. Polishing was conducted using cloths mounted on rotating wheels with synthetic diamond paste. Water lubrication was applied during sample grinding and polishing. The parallelism tolerances of all opposed surfaces were strictly inspected using a square.

### 2.2. DIC System Preparation

In preparation for the DIC measurements, the specimens were first painted white and then a random black spray pattern was applied on the surface. The DIC system used for image acquisition was a GOM ARAMIS 4M 2D DIC system manufactured by GOM in Braunschweig, Germany. The system consisted of a 4 Megapixel CMOS camera and a 35 mm lens. The image size was 2352 × 11,728 pixels^2^ and the acquisition frequency was 1 Hz. The recording frequency was set at 1 frame per second. The axial stress was obtained from the applied load divided by the specimen cross-sectional area.

The data collected was post-processed using the GOM ARAMIS 6.3.0 software provided by GOM mbH from Braunschweig, Germany to obtain the displacement and strain fields of the specimen surfaces. In order to avoid errors coming from the vicinity of the specimen edges, a rectangular central area of the size of 10 × 10 mm^2^ was selected as the region of interest, in which the displacement field and strain data were measured to represent the overall deformation of the specimen. According to Reu [23], the subset size was selected as 21 × 21 pixels^2^ to cover enough pattern features. The step size of 9 × 9 pixels^2^ was chosen to ensure sufficient spatial resolution. The virtual strain gauge (VSG) approach was used to calculate the strain.

### 2.3. Compression Test

**Figure 2:**
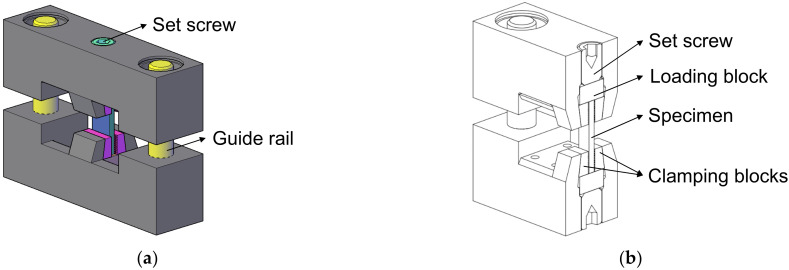

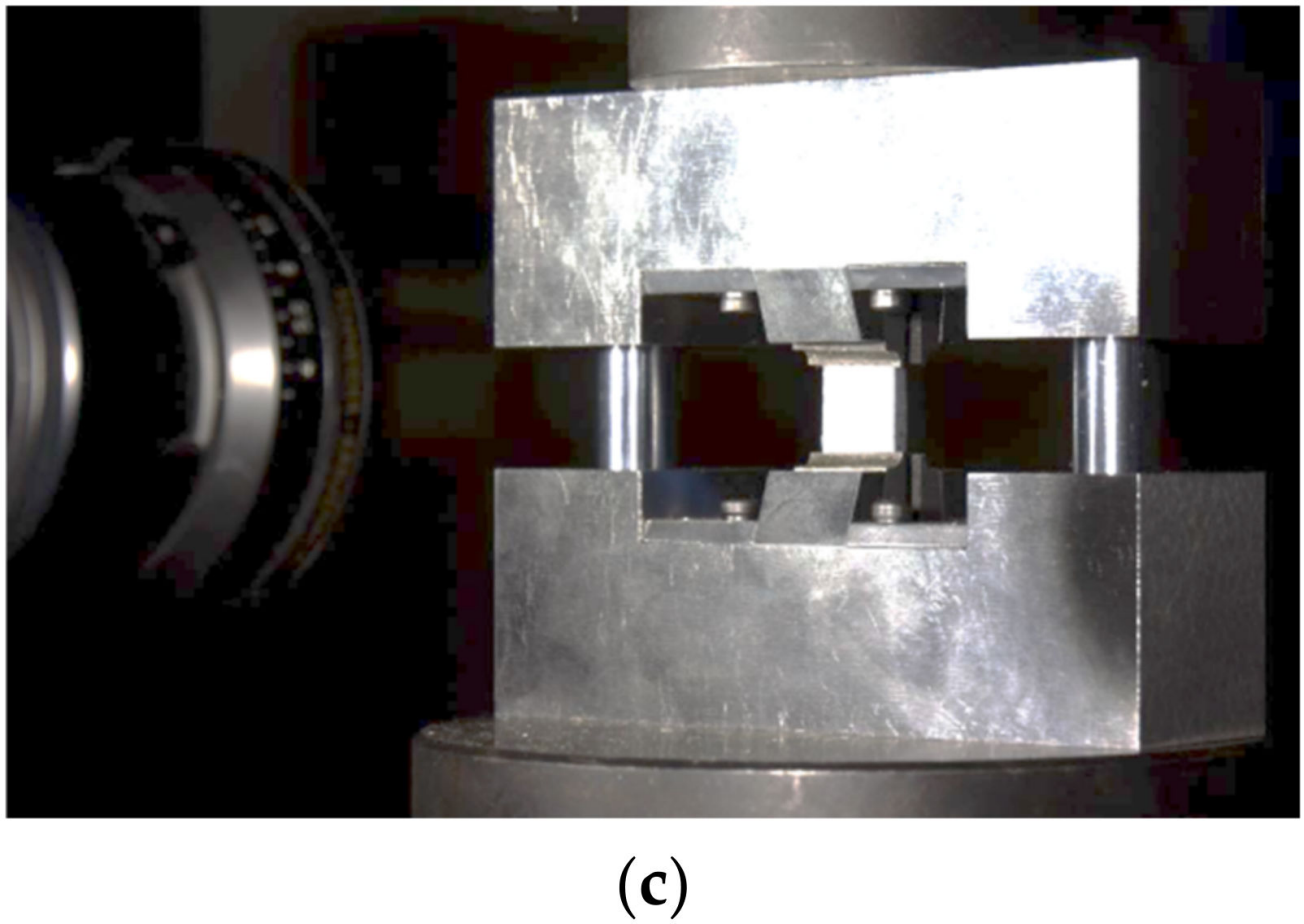
(**a**) The off-axis compression test fixture. (**b**) Sectional view of the off-axis compression test fixture. (**c**) The off-axis compression experimental setup.

To ensure stable loading to the small size specimens and eliminate the potential off-axis bending along the longitudinal direction, a special fixture was designed, as shown in Figure 2. When conducting the off-axis compression test, the specimen was placed in the centre of the fixture with the top and bottom ends supported by two loading blocks. The gripping areas of the specimen were clamped by two pairs of wedged clamping blocks to prevent the specimen from buckling and provide compressive loading through friction between the surfaces. The clamping blocks also provide lateral support to prevent specimen slipping. However, excessive gripping forces could cause specimen gripping area collapse and the specimen being over-constrained in the transverse direction. Hence, set screws were used to control the clamping forces to provide enough lateral support to the specimen while not introducing additional restraints.

The compression tests were carried out using a DDL100 electronic universal testing machine manufactured by Sinotest Equipment Co, Ltd in Changchun, China. A 100 kN load cell was used. The fixture was settled between the upper and lower compression platens, which were self-adjusted to be parallel to each other via a pair of contact spherical surfaces in the upper platen set. To ensure a quasi-static loading condition and to capture enough deformation images before failure for DIC analysis, the experiments were conducted under displacement control at a loading rate of 0.5 mm/min. The in-plane strain fields of the specimen were captured and determined by means of the digital image correlation (DIC) method. The strain gauges were also attached onto the back surface of the specimens to monitor the axial strain.

## 3. Results and Discussion

### 3.1. Failure Modes

**Figure 3:**
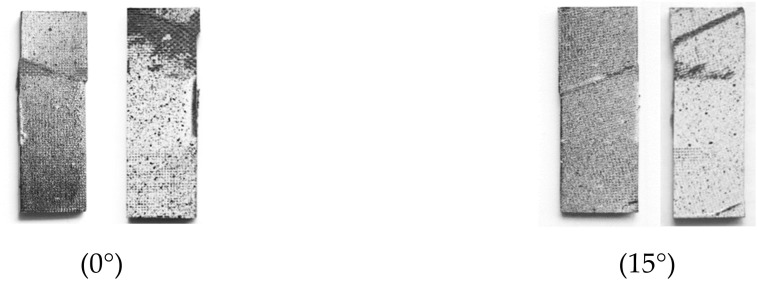

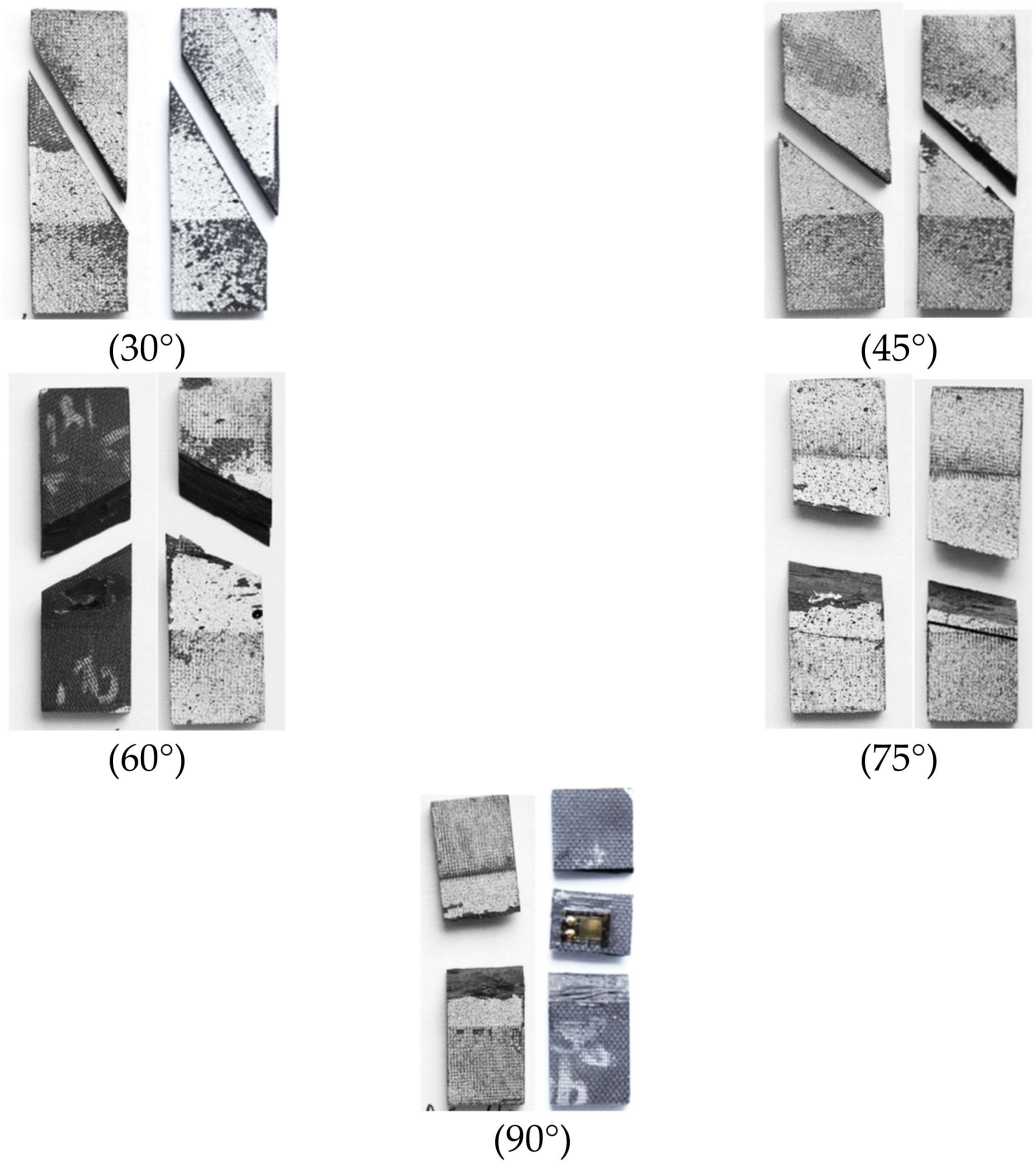
The failure modes of different specimens under compressive loads.

Figure 3 illustrates the failure modes of different specimens under compressive loading. For each specimen, the photographs of the front and back surfaces were taken to evaluate the failure mechanisms. No splitting and end collapse occurred in the 0° and 15° specimens, which indicates that the current test fixture can prevent the specimens from fibre splitting and end collapse. Hence, accurate compressive strengths of the off-axis specimens can be obtained through this testing method. In the case of the 30° off-axis angle, the fracture surface appeared to be scarcely slanted to the through thickness direction, indicating the existence of an in-plane shear failure mode, also referred to as splitting in some literature. A closer inspection of the fractured specimens revealed that kink-bands developed from the loading ends of the 0° and 15° specimens. In contrast, no sign of fibre kinking was found in the other specimens. This observation suggests that the transition from kinking to splitting occurred between 15° and 30°.

For the specimens with 45°, 60°, and 75° off-axis angles, the compression-shear mixed mode matrix failure was witnessed in the fracture surfaces developed along the fibre direction. For transverse compression (90°), specimens were normally separated into several pieces.

### 3.2. Mechanical Responses

**Figure 4:**
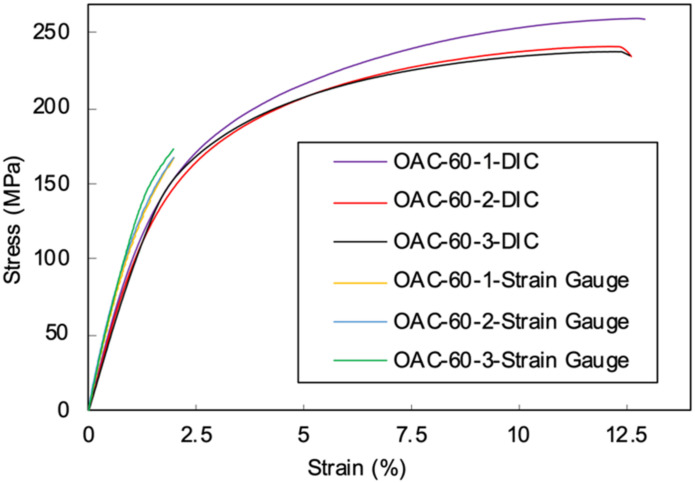
A comparison of the strain data collected from the DIC and strain gauge for the 60° off-axis compression test.

To evaluate the accuracy of the data collected by DIC, the stress–strain curves of specimens with 60° off-axis angles obtained from the strain gauges and DIC measurements were plotted together in Figure 4. A comparison of the two methods showed that the results from the strain gauges were slightly smaller. This is reasonable since the measuring grids of a strain gauge was mounted on a carrier foil and then attached to the specimen surface using glue; this insertion between the measuring grids and specimen surface can cause errors in the evaluated strain fields.

**Figure 5:**
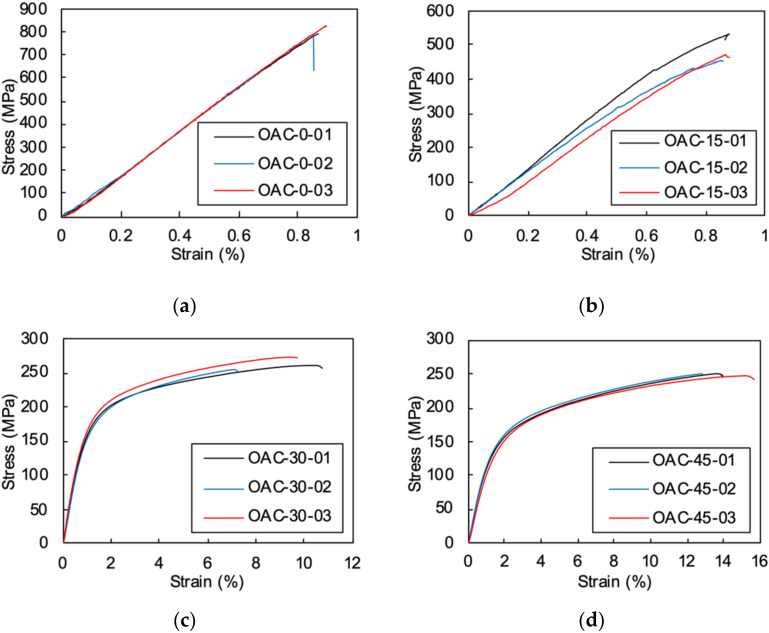

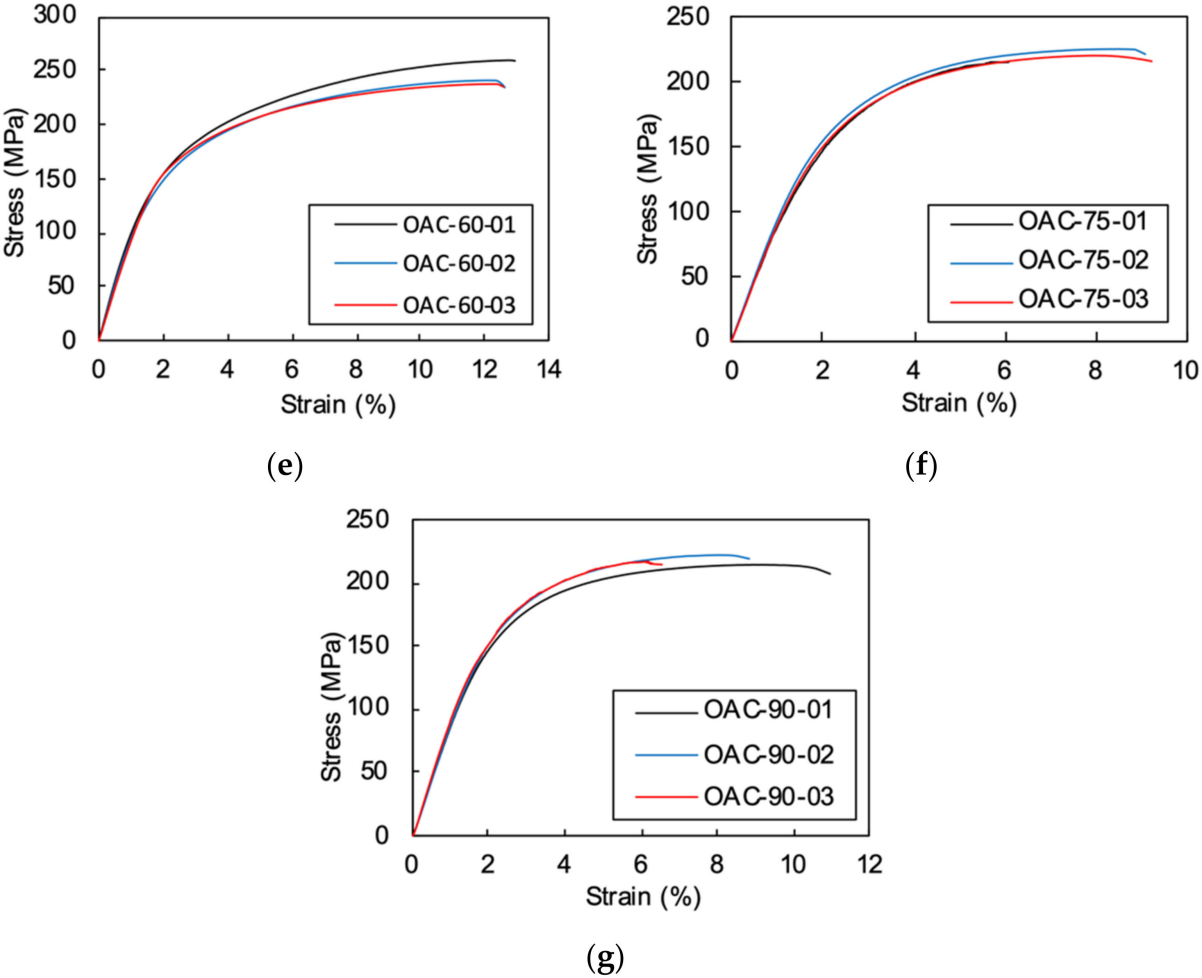
The stress-strain curves of the off-axis specimens under compression: (**a**) 0° axial compression; (**b**) 15° off-axis compression; (**c**) 30° off-axis compression; (**d**) 45° off-axis compression; (**e**) 60° off-axis compression; (**f**) 75° off-axis compression; (**g**) 90° transverse compression.

Typical stress–strain responses for different off-axis specimens are plotted in Figure 5. It can be seen that except for the 0° and 15° off-axis specimens, all of the other specimens exhibited apparent nonlinear deformation before failure. The mechanical response of 0° unidirectional compression is linear until final failure, all three specimens show good correlation. However, the 15° off-axis compression stress-strain relationships show obvious variation, a slight nonlinearity was observed in the curves. The maximum average strains of the 0° and 15° specimens were similar, while the average strength of the axial compression specimens was 350 MPa higher than in the 15° off-axis compression specimens.

For the 30° to 90° off-axis compression specimens, the initial moduli were linear until the strain hardening stages emerged. Final failures were reached after a significant plastic deformation stage. The 45° off-axis specimen had the highest failure strain of 14% while the 75° off-axis compression strain was around 9%. Compared to the thermoset composites [24], the thermoplastic composite exhibited prominent plastic deformation and much higher failure strains.

### 3.3. Fractography

Although a vast number of fractography investigations have been carried out for thermoset composites [25], few research has emphasised thermoplastic composites [26,27]. Some researchers have conducted fractography studies of thermoplastic composites under mode I and II fractures. The plastic behaviour of the thermoplastic matrix and its interaction with fibres during compressive/shear combined loadings still need fractographical analysis to reveal the failure mechanism.

**Figure 6:**
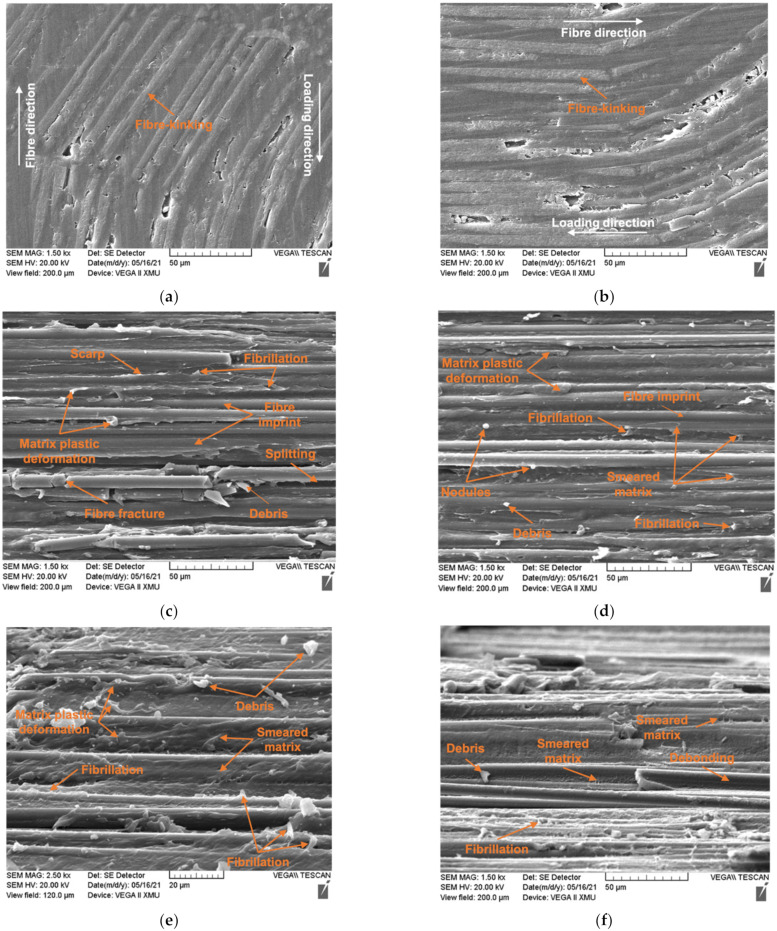

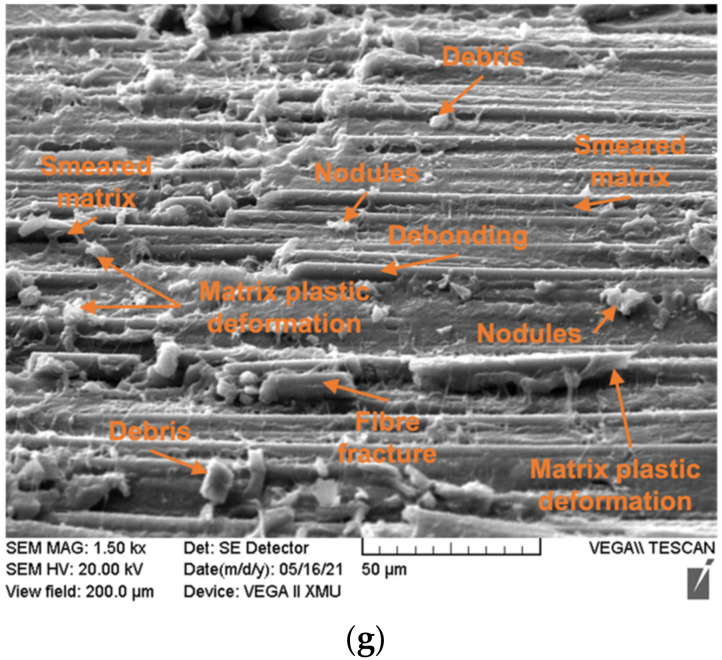
The SEM images of the fracture surfaces of the on-axis and off-axis specimens under compressive loads: (**a**) 0° axial compression; (**b**) 15° off-axis compression; (**c**) 30° off-axis compression; (**d**) 45° off-axis compression; (**e**) 60° off-axis compression; (**f**) 75° off-axis compression; (**g**) 90° on-axis transverse compression.

The tested specimens were inspected using a TESCAN VEGA II XMU scanning electron microscope (SEM) manufactured by TESCAN ORSAY HOLDING, Brno-Kohoutovice, Czech Republic. The SEM observations were conducted on all tested specimens at the in-plane direction, which was perpendicular to the thickness direction. Due to the different scales of the fracture surface, the SEM images were taken at various magnifications and are shown in Figure 6.

**Figure 7:**
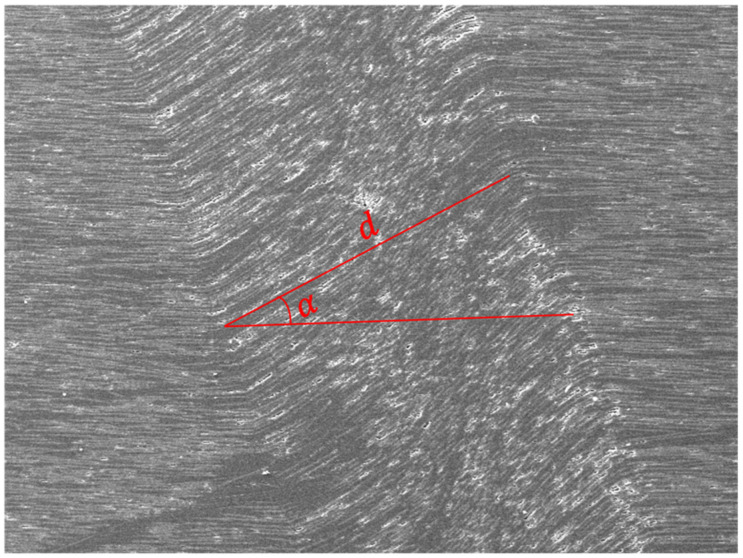
Measurement of the kink-band width and incline angle.

A clear fibre kink-band could be identified for the 0° and 15° off-axis specimens, indicating a thoroughly developed fibre kinking failure mechanism. From the 15° off-axis specimen image, severe fibre buckling without fibre fracture at the boundary of the kink-band could be seen. Fibre kinking in both the in-plane and out-of-plane directions were observed, indicating a three-dimensional failure mode. The kink-bands of the 0° and 15° specimens shared the same pattern. Generally, with sufficient lateral support from the matrix, fibres will fracture under shear stress instead of micro-buckling [1]. The ductile behaviour of the thermoplastic matrix often exhibited large plastic deformation and reduced lateral support to the fibres, which then triggered micro-buckling. Therefore, the scale of the kink band can be related to the mechanical parameters of the matrix. Compared to the thermoset composites, a longer kink band length implies a relatively ductile matrix. The parameters that control the fibre kinking formation can be measured, which are the kink-band width d and kink-band incline angle α, as shown in Figure 7. The measured average kink-band width and incline angle were 911.96 μm and 27.28°, respectively.

For the 30° off-axis compression, failure was dominated by the matrix fracture. A parabolic fracture pattern existed in the PEEK matrix region, indicating that the fracture grew from the apex toward the enclosed area of the parabola. The fibre was fractured and debris was generated due to the compressive loading along the fibre direction. Matrix plastic deformation caused by the shear force indicates the ductile damage mechanism of thermoplastic matrix. The ductile thermoplastic matrix draws on the interface and peels away the resin, resulted in debonding and nodules over the fibres [28]. Voids were nucleated, grew, and coalesced under high plastic deformation and resulted in fibrillation [29]. With the combined compressive stress, the shear fractured surface was abraded and showed a smeared morphology. The shearing of the matrix caused multiple micro-cracks inside the material. The micro-cracks continued to evolve until they coalesced. Scarps were generated in the coalescence locations.

The SEM inspection of the 45° specimens showed prominent matrix plastic deformation, indicating the plastic shear damage mode of the matrix. Additionally, fibrillation induced by noticeable plasticity could be spotted. The higher magnification SEM images of the 45° off-axis compression fracture surface showed interface debonding with nodules on the surface that were caused by the ductile drawing of the thermoplastic resin. Surface debris was produced as a result of the matrix shear/compression mixed failure.

On the fracture surface of the 60° off-axis specimens, fibre splitting and fractured short fibres were generated by the compression/shear combined stresses. A closer inspection at the fractured surface showed fibrillations and a smeared matrix, suggesting void deformation and a coalescence mechanism under a high-level of plastic deformation. Ductile drawing on the surface of the fibres was left due to the slow crack growth rate. Debris on the fracture surface was generated during the fracture of the matrix.

The failure modes of the 75° and 90° off-axis compression tests showed similar features. Many fibre fractures and splitting were produced on the fracture surface. The matrix exhibited highly plastic deformation and were drawn from the fibre surface. Debris and nodules were left on the surface of the fibres and matrix after the compression dominated fracture.

Compared to the shear/compression fracture of brittle thermoset epoxies, in which the dominant fracture feature is the cusp formation mechanism with companion cleavages, riverlines, and textured microflows, no shear cusp was detected for the thermoplastic composites. The tough thermoplastic material had a higher fracture energy and more ductility than the thermosets, leading to plastic deformation and void coalescence dominated failure mechanisms. Riverlines, which are often seen in brittle fractured polymers, were also not found. Therefore, it is hard to deduce the fracture direction of the thermoplastic matrix.

Moreover, for the semi-crystalline thermoplastic PEEK matrix, the fibre surface can induce nucleation to form crystallization. The PEEK/AS4 composite would generate a transcrystallization interphase. As can be seen in Figure 6c–g, little fibre/matrix debonding was observed. This indicates that the transcrystallization improved the bonding strength between the fibres and the matrix. Although debonding failure was often observed in the thermoset matrix composites, the failure modes of the thermoplastic composites were mainly matrix plastic deformation and cracking.

### 3.4. Visualization of Displacements and Strains

Full field displacements and strains of the region of interest were analysed using the DIC method. To eliminate errors from the free edges and clamping, areas near the gripping lines and edges were not included.

**Figure 8:**
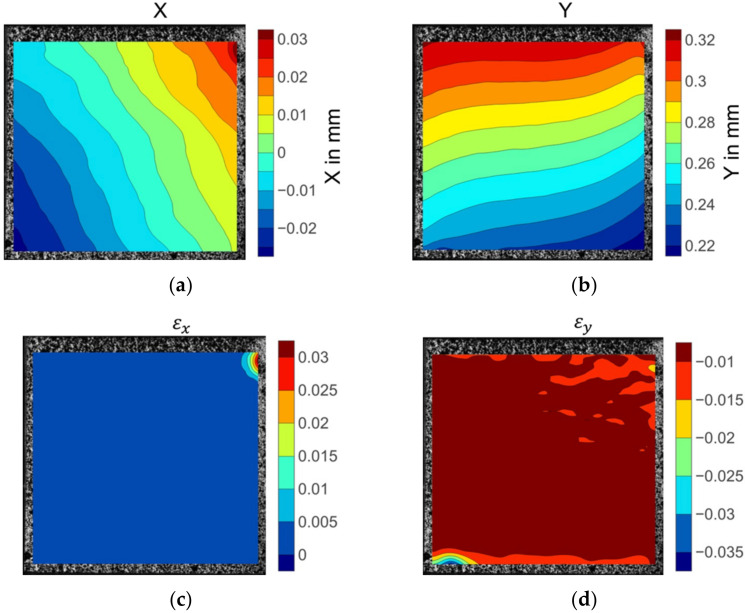
The displacement and strain field contour plots of the 0° axial compression specimen: (**a**) Displacement in the X direction; (**b**) displacement in the Y direction; (**c**) strain in the X direction; (**d**) strain in the Y direction.

Figure 8 illustrates the displacement and strain fields of the 0° on-axis compression specimen before fibre kinking failure. The X direction displacement distribution in Figure 8a was not parallel to the fibre direction, showing a nonuniform deformation across the width of the specimen. The longitudinal displacements were evenly placed over the specimen width, as shown in Figure 8b. The strain fields in both the x and y directions were consistent in the centre area of the specimen (see Figure 8c,d). The deep red region in the upper right corner of the X direction strain nephogram $\varepsilon_{x}$ (see Figure 8c) indicates severe strain concentration. However, no damage was discovered in the strain concentration position when inspecting the specimen after failure. Unlike the epsilon x field distribution, slightly higher epsilon y strain values were observed in the upper right side of the strain field. Significant strain concentration emerged in the left bottom area of the contour plot of $\varepsilon_{y}$ strain fields, as shown in the light coloured region in Figure 8d. The extreme levels of strain coincided with the region where fibre kinking occurred.

**Figure 9:**
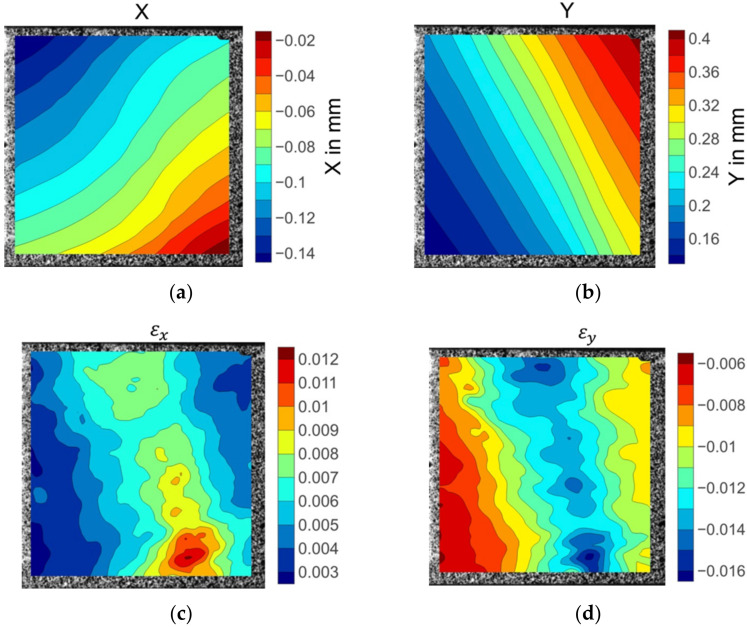
The displacement and strain field contour plots of the 15° off-axis specimen: (**a**) Displacement in the X direction; (**b**) displacement in the Y direction; (**c**) strain in the X direction; (**d**) strain in the Y direction.

Figure 9 shows the axial and transverse displacement and strain fields of the 15° off-axis specimen prior to failure initiation. The displacement field contour plots showed clear patterns before failure. The transverse displacement gradient changed perpendicular to the fibre direction, while the axial displacement gradient varied parallel to the fibres. The strain fields were not evenly distributed over the specimen. The transverse strain fields demonstrated a high level of strain concentration in the area where fibre kinking occurred. The contour plot of the axial strain fields indicates high strain gradient variation along the width of the specimen.

**Figure 10:**
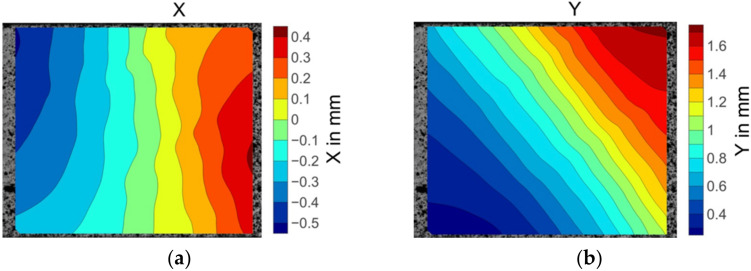

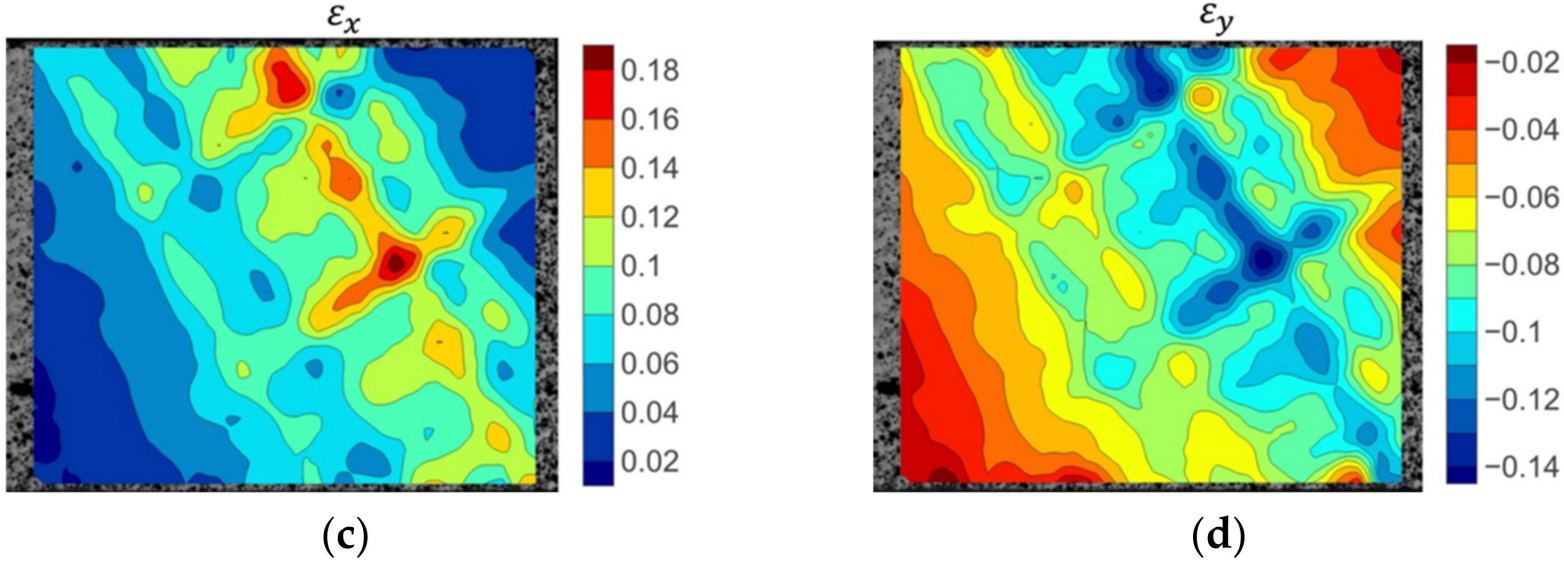
The displacement and strain field contour plots of the 30° off-axis specimen: (**a**) Displacement in the X direction; (**b**) displacement in the Y direction; (**c**) strain in the X direction; (**d**) strain in the Y direction.

The illustrated displacement fields of the 30° off-axis specimen in Figure 10a,b indicate that the displacement gradients were consistent. However, the strain was not evenly distributed. The area in the deep red colour at the centre right in Figure 10c illustrates the highest $\varepsilon_{x}$ strain values recorded by the DIC system. An extreme level of the $\varepsilon_{y}$ strain was also identified close to the transverse strain concentration location. These strain concentrations represent the maximum matrix deformation and thereby indicate the matrix damage initiation site.

**Figure 11:**
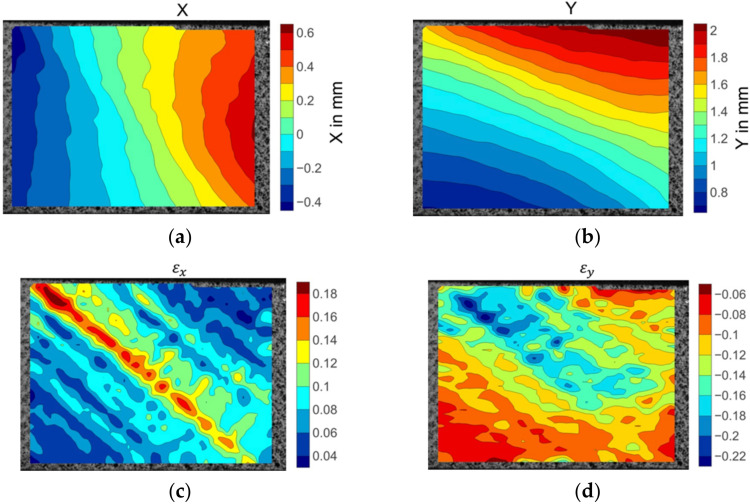
The displacement and strain field contour plots of the 45° off-axis specimen: (**a**) Displacement in the X direction; (**b**) displacement in the Y direction; (**c**) strain in the X direction; (**d**) strain in the Y direction.

Figure 11 depicts the displacement and strain fields before the matrix cracking failure of a 45° off-axis specimen. The displacement fields were conformably distributed along the fibre direction. The strain fields showed inconsistent strain gradients. The strain concentration points were scattered on the surface. High $\varepsilon_{x}$ strain value points were distributed, which were aligned as the red coloured regions in Figure 11c. This strain concentration route corresponds to the matrix cracking route. The peak values of $\varepsilon_{y}$, strain marked in a deep blue colour in Figure 11d, coincided with the locations of the highest $\varepsilon_{x}$ strain points. This could be induced by the local matrix micro-cracking damage.

**Figure 12:**
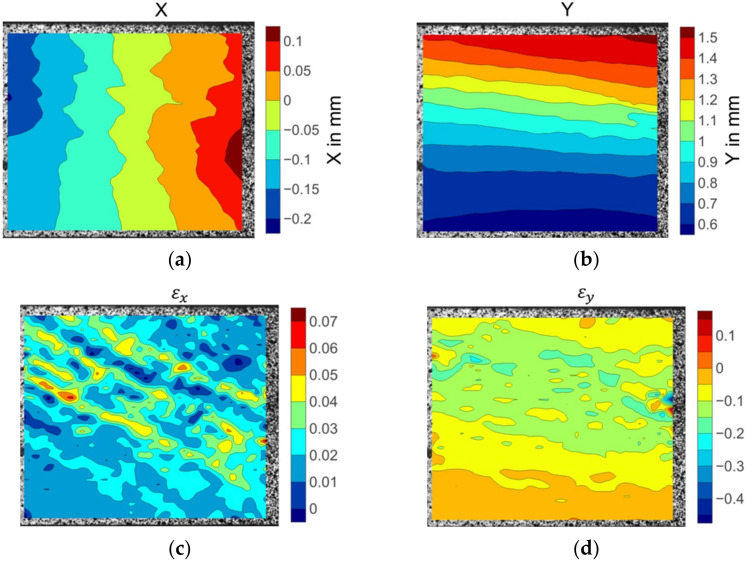
The displacement and strain field contour plots of the 60° off-axis specimen: (**a**) Displacement in the X direction; (**b**) displacement in the Y direction; (**c**) strain in the X direction; (**d**) strain in the Y direction.

The displacement and strain fields of the 60° off-axis specimen is shown in Figure 12. High strain gradients along the fibre direction were captured prior to failure. Although some strain concentration areas coincided with the matrix cracking failure position, the specimen surface with no fracture also exhibited strain concentration.

**Figure 13:**
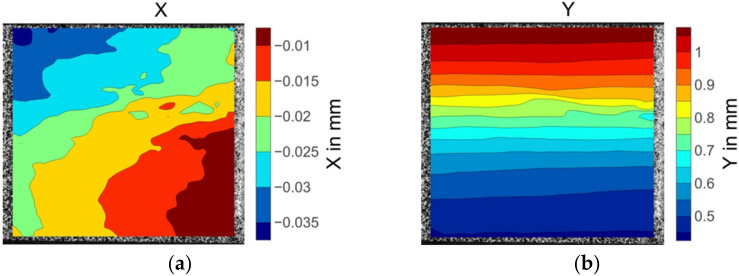

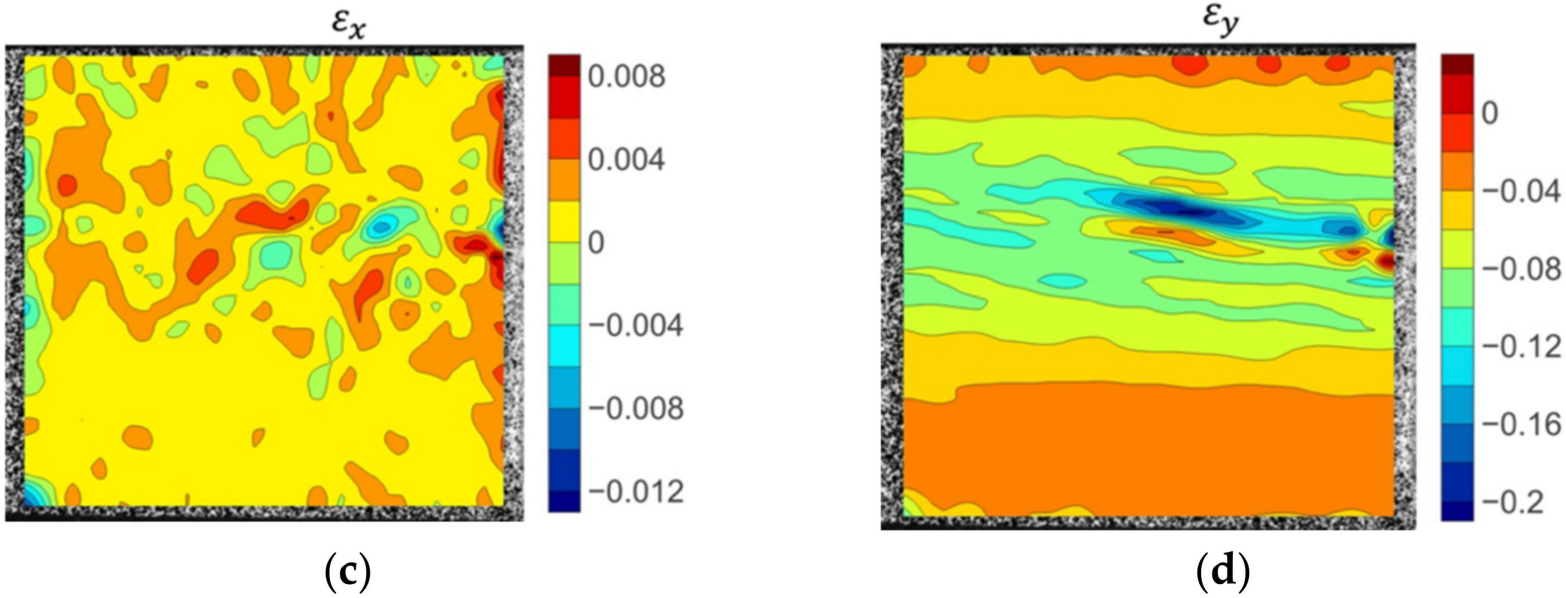
The displacement and strain field contour plots of the 75° off-axis specimen: (**a**)displacement in X direction; (**b**) displacement in Y direction; (**c**) strain in X direction; (**d**) strain in Y direction.

As the off-axis angle inclined to 75°, the prior to failure transverse displacement field shown in Figure 13 varied prominently. Large deformation gradients appeared in the bottom right location as shown in the deep red colour in Figure 13a. The $\varepsilon_{y}$ strain fields contained significant concentrations along the matrix fracture path. The severity of the strain concentration denotes the matrix damage accumulation level. The highest strain concentration level represents the failure initiation location. The $\varepsilon_{x}$ strain patterns showed local variation with the strain concentrations scattered over the specimen surface. The peak values coincided with the failure point.

**Figure 14:**
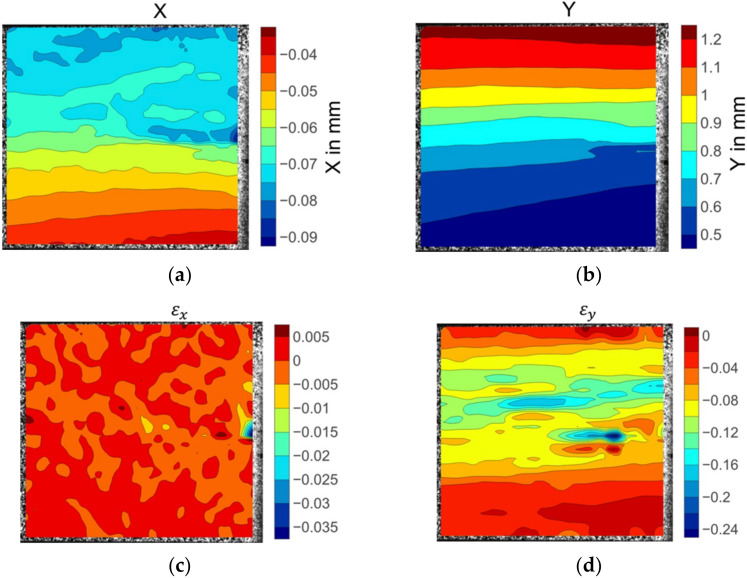
The displacement and strain field contour plots of the 90° on-axis transverse compression specimen: (**a**) Displacement in the X direction; (**b**) displacement in the Y direction; (**c**) strain in the X direction; (**d**) strain in the Y direction.

The displacement and strain fields of the 90° on-axis transverse compression specimen is shown in Figure 14. The specimen exhibited a consistent displacement arrangement. Apparent strain concentrations of $\varepsilon_{y}$ appeared in accordance with the final fracture site. Cracks originated from these peaks of strain values to form the fracture surface.

### 3.5. Failure Envelope

**Figure 15:**
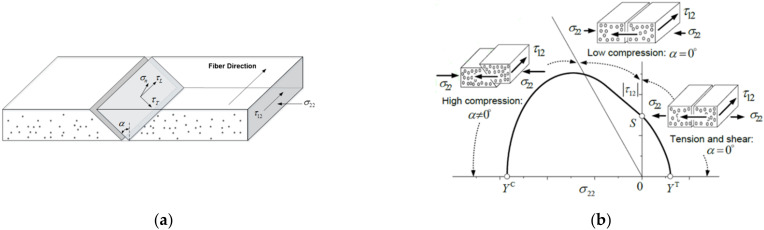
(**a**) The angle between the fracture surface and the through thickness direction and (**b**) the change in the fracture angle α under different stress conditions.

According to Puck and Schurmann [30], when the T300/914C carbon fibre reinforced epoxy unidirectional laminate is under transverse compression ($\sigma_{22}<0$) and in-plane shear, matrix cracks will initiate on a surface parallel to the shear direction and propagate along the fibre direction, as shown in Figure 15a. The angle α between the fracture surface and the through thickness direction varied under different stress states, as shown in Figure 15b. For most thermoset composites, as the value of $\left|\sigma_{22}\right|$ increases, the fracture surface angle α increased from 0° to 53°.

**Figure 16:**
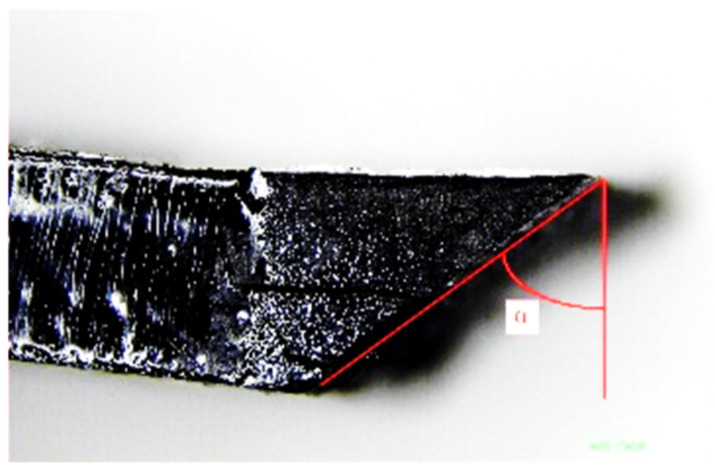
The fracture angles of the off-axis compression specimens.

**Figure 17:**
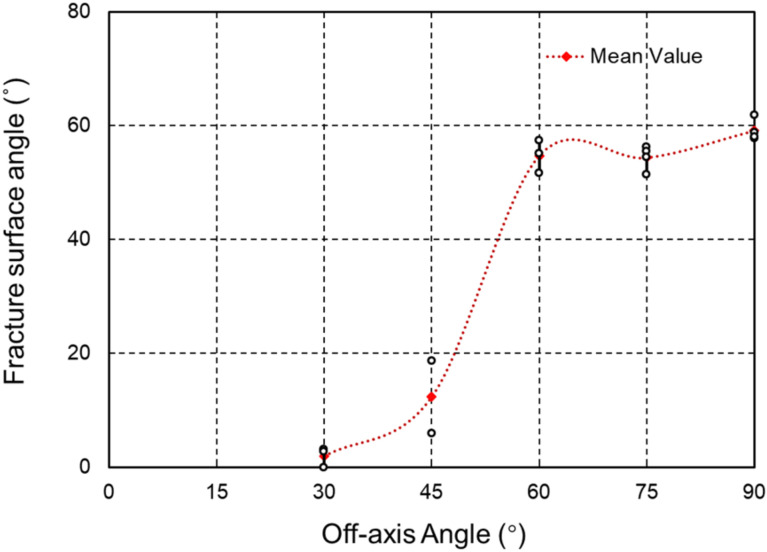
The fracture angles vs. the off-axis angles.

The failure modes of the CFRTP specimens that were tested also showed similar features of the fracture surface, as shown in Figure 16. Figure 17 shows the curve of the fracture angle vs. the off-axis angle. The angle α increased to 12.3° for the 45° off-axis specimens before zooming to 54.75°, and the maximum fracture surface angle was attained as 59.16° for the 90° compression case.

According to the Hashin failure criterion [31] for the plane stress condition, and combining the stress transformation equations, the values of $\sigma_{22}$ and $\tau_{12}$ at compressive failure for the off-axis unidirectional laminates are:

(1) $$\sigma_{22}=X\sin^{2}\theta$$

(2) $$\tau_{12}=X\sin\theta \cos\theta$$

where *X* is the strength upon failure:

(3) $$X=\frac{S_{L}Y_{C}}{\sin\theta \sqrt{S_{L}^{2}\sin^{2}\theta +Y_{C}^{2}\cos^{2}\theta }}$$

where $S_{L}$ is the in-plane shear strength obtained from the experiments; $Y_{C}$ is the transverse compressive strength; θ is the off-axis angle of the fibers; $\sigma_{22}$ and $\tau_{12}$ are the transverse normal stress and in-plane shear stress upon failure, respectively.

The LaRC05 theory is considered as one of the most accurate failure criteria according to the second World-Wide Failure Exercise (WWFE-II). This failure criterion is proposed as a physically-based model that characterises the failure modes of composites in three types: matrix failure, fibre compression failure, and fibre tension failure.

For the matrix failure mode (MF):

(4) $${\left(\frac{\tau_{T}}{S_{T}^{is}-\eta_{T}\sigma_{n}}\right)}^{2}+{\left(\frac{\tau_{L}}{S_{L}^{is}-\eta_{L}\sigma_{n}}\right)}^{2}+{\left(\frac{❬\sigma_{n}❭}{Y_{T}^{is}}\right)}^{2}=1$$

where $❬\sigma_{n}❭=\left(\sigma_{n}+\left|\sigma_{n}\right|\right)/2$; $S_{T}^{is}$, $S_{L}^{is}$, and $Y_{T}^{is}$ are the in-situ transverse shear strength, in-situ longitudinal shear strength, and in-plane transverse tension strength, respectively. The fracture surface stress components $\sigma_{n}$, $\tau_{T}$, and $\tau_{L}$ are given by Puck [30]:

(5) $$\left\{\begin{array}{c} \sigma_{n}=\frac{\sigma_{2}+\sigma_{3}}{2}+\frac{\sigma_{2}-\sigma_{3}}{2}\cos2\alpha +\tau_{23}\sin2\alpha \\ \tau_{T}=-\frac{\sigma_{2}-\sigma_{3}}{2}\sin2\alpha +\tau_{23}\cos2\alpha \\ \tau_{L}=\tau_{12}\cos\alpha +\tau_{31}\sin\alpha \end{array}\right.$$

where α is the fracture surface angle that can be determined numerically or experimentally, $\alpha_{0}$ represents the fracture surface angle of transverse compression of unidirectional plates.

For the fibre compression kinking failure mode:

(6) $${\left(\frac{\tau_{23}^{m}}{S_{T}^{is}-\eta_{T}\sigma_{2}^{m}}\right)}^{2}+{\left(\frac{\tau_{12}^{m}}{S_{L}^{is}-\eta_{L}\sigma_{2}^{m}}\right)}^{2}+{\left(\frac{❬\sigma_{2}^{m}❭{}_{+}}{Y_{T}^{is}}\right)}^{2}=1,\;\sigma_{1}\leq -\frac{X_{c}}{2}$$

where $\eta_{T}$ and $\eta_{L}$ are the transverse shear and longitudinal shear friction coefficient, and are defined as:

(7) $$\eta_{T}=-\frac{1}{\tan2\alpha_{0}},\;\eta_{L}=S_{L}\frac{\eta_{T}}{S_{T}}$$

where $S_{L}$ and $S_{T}$ are the longitudinal and transverse shear strength of the unidirectional composites on the fracture surface:

(8) $$S_{T}=\frac{Y_{C}}{2\tan\alpha_{0}}$$

where $Y_{C}$ is the transverse compression strength and $\alpha_{0}$ is the fracture plane angle to the thickness direction under transverse compression.

For the fibre compression splitting failure mode:

(9) $${\left(\frac{\tau_{23}^{m}}{S_{T}^{is}-\eta_{T}\sigma_{2}^{m}}\right)}^{2}+{\left(\frac{\tau_{12}^{m}}{S_{L}^{is}-\eta_{L}\sigma_{2}^{m}}\right)}^{2}+{\left(\frac{❬\sigma_{2}^{m}❭{}_{+}}{Y_{T}^{is}}\right)}^{2}=1,-\frac{X_{c}}{2}\leq \sigma_{1}\leq 0$$

For the fibre tension failure, the maximum stress criterion was adopted:

(10) $$\frac{❬\sigma_{1}❭{}_{+}}{X_{T}}=1$$

where $X_{T}$ is the longitudinal tension strength.

The in situ strengths for the unidirectional composites are calculated as:

(11) $$\left\{\begin{array}{c} Y_{T}^{is}=Y^{T}\\ S_{Y}^{is}=S^{Y}\\ S_{T}^{is}=Y_{C}\cos\alpha_{0}\sin\alpha_{0}+\frac{\cos\alpha_{0}}{\tan2\alpha_{0}} \end{array}\right.$$

where $Y_{C}$ is the transverse compression strength; $Y^{T}$ is the transverse tension strength; and $S^{Y}$ is the shear strength. The value of $\eta_{L}$ can be deduced from the off-axis compression test. All material properties required for the numerical simulation using LaRC05 criterion are listed in Table 2.

**Figure 18:**
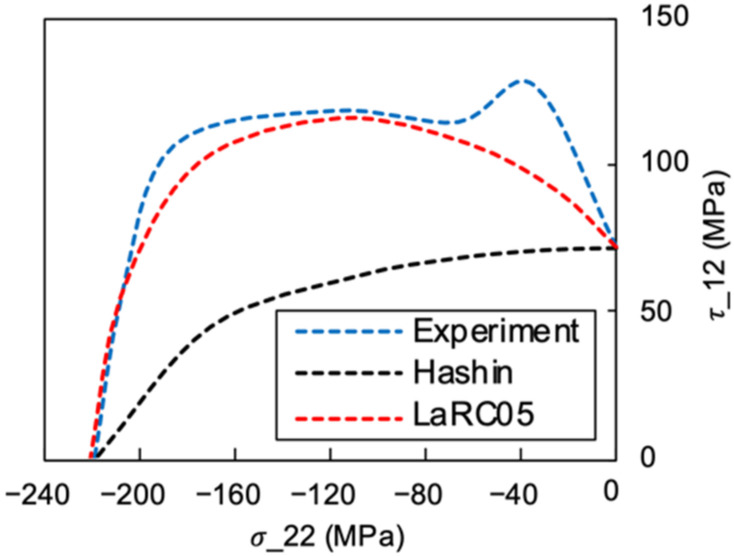
The off-axis compression failure envelope.

The $\sigma_{22}-\tau_{12}$ failure envelope in the second quadrant based on LaRC05 and the Hashin failure criteria are plotted in Figure 18, and were compared to the results obtained from the off-axis tests. It can be seen from the experimental results that the transverse compression led to the increased shear strength, which agrees with Puck’s theory. The predictions based on the Hashin failure criterion were over conservative with regard to the experimental results. In contrast, the LaRC05 failure criterion [32], which takes the in situ effects into consideration and includes the fibre-kinking mechanism in the fibre failure modes, has shown a more accurate prediction than Hashin’s criteria for the compression-shearing coupling scenarios. However, underestimation still existed for the strength predictions of the LaRC05 criterion when the off-axis angle was under 15°. The reason is that the LaRC05 criterion accurately predicts fibre-kinking failure when the matrix is relatively brittle. The thermoplastic PEEK matrix showed a much higher toughness and ductility compared to the thermoset polymers. Hence, the thermoplastic matrix provided a better support to the fibres and delayed the initiation of fibre-kinking damage. In order to apply the LaRC05 criterion to the failure prediction of the thermoplastic PEEK matrix composites, the fibre-kinking theory and criterion should be modified to include the ductile damage mechanism of the thermoplastic material.

## 4. Conclusions

An experimental investigation of the unidirectional PEEK/AS4 carbon fibre reinforced thermoplastic composites under off-axis compression was conducted using the DIC technique. The conclusions can be drawn as follows.

1. With the new designed off-axis compression test fixture, the stress concentration, fibre splitting, and end collapse could be avoided during the off-axis compression tests;
2. The material matrix exhibited evident nonlinearity under a mixed shear-compression stress state, so a constitutive model should be proposed in further study;
3. Fibre kinking was observed in the specimens with small off-axis angles. The trend of the fracture modes and the fracture angles through the thickness direction vs. the off-axis angles were also observed and discussed. The shear matrix dominated the failure as the angle increased and a high level of plastic deformation of the thermoplastic matrix was observed;
4. The compression failure envelope for different shear–compression combinations predicted based on the Hashin criterion was too conservative compared to the experimental results, while the LaRC05 criterion made excellent predictions when the off-axis angle was larger than 15°.

## Figures and Tables

**Table 1:** The properties of the PEEK/AS4 prepreg.

	PEEK/AS4
Matrix system	Polyetheretherketone (PEEK)
Fibre	AS4 carbon fibre
Fibre volume fraction	34%
Overall density	1.46 g/cm^3^
Moulding	Hot press moulding
Thickness	0.14 mm

**Table 2:** The material parameters of PEEK/AS4.

$E_{11}$ **(GPa)**	$E_{22}$ **(GPa)**	$E_{33}$ **(GPa)**	$G_{12}$ **(GPa)**	$G_{23}$ **(GPa)**	$G_{13}$ **(GPa)**	$\upsilon_{12}$	$\upsilon_{13}$	$\upsilon_{23}$	$X_{T}$ **(MPa)**
133	9.08	9.08	4.31	3.44	4.31	0.3	0.3	0.5	2001
$X_{C}$ **(MPa)**	$Y_{T}$ **(MPa)**	$Y_{C}$ **(MPa)**	$S_{L}$ **(MPa)**	$S_{T}$ **(MPa)**	$\varphi_{0}$	$\alpha_{0}$	$\eta_{L}$	$\eta_{T}$	
816	104	201	72.4	65.6	4.5°	59°	0.59	0.54	

## Data Availability

Data available in a publicly accessible repository.
